# Achieving 21% External Quantum Efficiency for Nondoped Solution‐Processed Sky‐Blue Thermally Activated Delayed Fluorescence OLEDs by Means of Multi‐(Donor/Acceptor) Emitter with Through‐Space/‐Bond Charge Transfer

**DOI:** 10.1002/advs.201902087

**Published:** 2020-02-08

**Authors:** Xujun Zheng, Rongjuan Huang, Cheng Zhong, Guohua Xie, Weimin Ning, Manli Huang, Fan Ni, Fernando B. Dias, Chuluo Yang

**Affiliations:** ^1^ Renmin Hospital of Wuhan University Hubei Key Lab on Organic and Polymeric Optoelectronic Materials Department of Chemistry Wuhan University Wuhan 430072 P. R. China; ^2^ Shenzhen Key Laboratory of Polymer Science and Technology College of Materials Science and Engineering Shenzhen University Shenzhen 518060 China; ^3^ Department of Physics Organic Electroactive Materials Group Durham University Durham DH1 3LE UK

**Keywords:** multi‐(donor/acceptor), nondoped solution‐processed OLEDs, sky‐blue, thermally activated delayed fluorescence, through‐space/‐bond charge transfer

## Abstract

Although numerous thermally activated delayed fluorescence (TADF) organic light‐emitting diodes (OLEDs) have been demonstrated, efficient blue or even sky‐blue TADF‐based nondoped solution‐processed devices are still very rare. Herein, through‐space charge transfer (TSCT) and through‐bond charge transfer (TBCT) effects are skillfully incorporated, as well as the multi‐(donor/acceptor) characteristic, into one molecule. The former allows this material to show small singlet–triplet energy splitting (Δ*E*_ST_) and a high transition dipole moment. The latter, on the one hand, further lights up multichannel reverse intersystem crossing (RISC) to increase triplet exciton utilization via degenerating molecular orbitals. On the other hand, the nature of the molecular twisted structure effectively suppresses intermolecular packing to obtain high photoluminescence quantum yield (PLQY) in neat flims. Consequently, using this design strategy, T‐CNDF‐T‐*t*Cz containing three donor and three acceptor units, successfully realizes a small Δ*E*_ST_ (≈0.03 eV) and a high PLQY (≈0.76) at the same time; hence the nondoped solution‐processed sky‐blue TADF‐OLED displays record‐breaking efficiency among the solution process‐based nondoped sky‐blue OLEDs, with high brightness over 5200 cd m^−2^ and external quantum efficiency up to 21.0%.

## Graphical abstract

A novel multi‐(donor/acceptor) thermally activated delayed fluorescence (TADF) molecule with through‐space/‐bond charge transfer is developed. Its nondoped solution‐processed sky‐blue organic light‐emitting diode (OLED) displays high performance with an external quantum efficiency (EQE_max_) up to 21.0%, which represents the record‐breaking efficiency among the solution process‐based nondoped sky‐blue OLEDs.

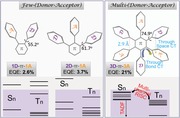

Owing to their superior display quality and flexibility, organic light‐emitting diodes (OLEDs) have been leading the innovation in flat panel displays and lighting applications. Unfortunately, the spin statistics make OLEDs using conventional organic fluorescent emitters very inefficient, with an upper limit to their internal quantum efficiency (IQE) of 25% due to the fact that only one‐quarter of the excitons are singlets.^1^ However, thermally activated delayed fluorescence (TADF) emitters, pioneered by C. Adachi, can utilize the up‐conversion from triplet (T_1_) to singlet (S_1_) states through reverse intersystem crossing (RISC). Thus, nearly 100% IQE could be obtained in noble‐metal‐free electroluminescence (EL) devices.^2^, ^3^, ^4^, ^5^, ^6^ Moreover, TADF materials would help to overcome the putative disadvantages of Ir or Pt‐containing systems,^7^, ^8^, ^9^ such as the relatively high cost, scarce resources, and potential environmental problems.

To date numerous highly efficient TADF‐based OLEDs have been successfully reported,^10^, ^11^, ^12^, ^13^, ^14^, ^15^, ^16^, ^17^, ^18^, ^19^ including solution‐processed blue emitting devices.^20^, ^21^ Since this manufacturing technique is amenable to the industrialization of OLEDs, due to its natural distinctive advantages, such as the low‐cost, high processing efficiency, easy scalability, and better controlling of the doping concentration.^22^, ^23^ In 2018, Kaji and coworkers applied an adamantyl substitution strategy to access solution processable blue TADF‐OLEDs with external quantum efficiency (EQE) of up to 22%.^24^ However, these devices required the TADF material being used as a dopant in different hosts, which makes its manufacturing process too complicated, as well as rendering poor repeatability and stability. Therefore, this strategy is not favorable for large‐scale industrial production. Earlier, in 2017, Lu's group reported a nondoped solution‐processed blue TADF‐OLED, but the efficiency was less than 20%.^25^ Highly efficient OLEDs, with blue or even sky‐blue emission, based on TADF nondoped layers, and fabricated by solution‐processed methods are still very rare, and remain a great challenge.

Herein, we report a charge‐transfer‐featured TADF emitter, namely T‐CNDF‐T‐*t*Cz (vide **Figure** **1**a), with three donor (D) and three acceptor (A) units, in which di‐*tert*‐butylcarbazole (*t*Cz), benzene (ph), and difluorocyanobenzene (CNDF) act as an electron donor, a π‐bridge, and an electron acceptor, respectively. Due to the fact that the D and A units in T‐CNDF‐T‐*t*Cz are connected to the central benzene bridge by alternating arrangement, the spatial vertical distance between D and A was decreased while the twisted angles of D (or A) with benzene group were increased. As expected, on one hand, the alternating arrangement of D–A units endows T‐CNDF‐T‐*t*Cz the coexistence of through‐space charge transfer (TSCT) and through‐bond charge transfer (TBCT) effects, resulting in a small Δ*E*_ST_ and high photoluminescence quantum yield (PLQY).^26^ On the other hand, the multi‐(donor/acceptor) structure in T‐CNDF‐T‐*t*Cz promotes spin‐vibronic mixing among the multiple excited states, which is crucial to the efficient multichannel RISC process.^10^ Remarkably, its highly twisted structure could suppress the intermolecular π–π stacking, leading to reduced fluorescence quenching in condensed state.^27^, ^28^ Therefore, the nondoped solution‐processed sky‐blue TADF‐OLED displays high performance with a brightness (*B*_max_) over 5200 cd m^−2^, and maximum external quantum efficiency (EQE_max_) up to 21.0%, which represents the record‐breaking EL efficiency among the solution process‐based nondoped blue or sky‐blue devices. In contrast, the reference molecules with fewer donor/acceptor fragments, S‐CNDF‐S‐*t*Cz and S‐CNDF‐D‐*t*Cz (see Figure 1a), show less efficient RISC processes and lower PLQYs. Consequently, the corresponding devices render inferior performances. Our results indicated that such multi‐(donor/acceptor) TADF molecules combined with TSCT and TBCT effects should be promising TADF emitters, and may shed light on the molecular design to achieve high‐performance OLEDs.

**Figure 1:**
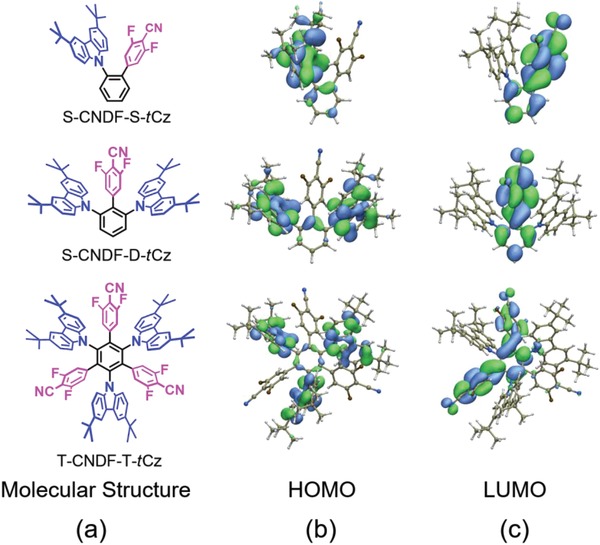
a–c) The molecular structures (a) and the HOMO (b), and LUMO (c) distributions of S‐CNDF‐S‐*t*Cz (top), S‐CNDF‐D‐*t*Cz (middle), and T‐CNDF‐T‐*t*Cz (bottom).

According to our design strategy, the donor *t*Cz and the acceptor CNDF are connected on the benzene ring by mutual *ortho*‐position, which generates an interchromophore packing with a distance less than 3.3 Å, the effective van der Waals distance. Hence, charge could be transferred through space and linker simultaneously in T‐CNDF‐T‐*t*Cz, achieving a balance between small Δ*E*_ST_ and high PLQY (*Φ*_PL_). Moreover, the multi‐(donor/acceptor) architecture could further enhance PLQY with the intermolecular stacking inhibited, and accelerate the RISC process due to the degenerated excited state. The design strategy was guided by time‐dependent density functional theory (TD‐DFT) based on the PBE0 functional and a def2‐SVP basis. As shown in Figure S1b in the Supporting Information, in the optimized structure of T‐CNDF‐T‐*t*Cz, the dihedral angles of *t*Cz and CNDF with the linker ph are 73.7^o^ and 56.2^o^, respectively, and the minimum spatial distance between D and A is less than 3.3 Å, suggesting a more effective intramolecular π–π interaction between donor and acceptor, i.e., TSCT, compared with the reference compounds (50.8^o^ and 47.8° for S‐CNDF‐S‐*t*Cz, and 57.0^o^ and 55.7° for S‐CNDF‐D‐*t*Cz, respectively).

In view of the frontier molecular orbital (FMO), the highest occupied molecular orbitals (HOMOs) of these molecules are mainly located on the *t*Cz units (Figure 1b), while the lowest unoccupied molecular orbitals (LUMOs) distribute in the CNDF units (Figure 1c). The spatial separated HOMO and LUMO may ensure that the emitters own the small Δ*E*_ST_s to guarantee a valid TADF. It is worth mentioning that the phenyl bridges are all involved in contributing their frontier orbitals, which suggests that charge can also be transferred through the linker directly, i.e., TBCT. The overlap of HOMO and LUMO either on phenyl bridge or in the stacking space could lead to TBCT/TSCT emission, respectively. The HOMO–LUMO overlaps contributed through space/through phenyl bridge are 0.22/0.05 for T‐CNDF‐T‐*t*Cz, 0.26/0.03 for S‐CNDF‐D‐*t*Cz, and 0.15/0.07 for S‐CNDF‐S‐*t*Cz. The proportions of TBCT/TSCT can be further characterized by integrating the transition density that is localized on/not on the benzene bridge. According to the calculations, the proportions of TSCT/TBCT in the S_1_ state of T‐CNDF‐T‐*t*Cz, S‐CNDF‐D‐*t*Cz, and S‐CNDF‐S‐*t*Cz are 77.2%/22.8%, 76.5%/23.5%, and 63.7%/36.3%, respectively.

In principle, T‐CNDF‐T‐*t*Cz have three quasi equivalent donor and acceptor, which could promote the RISC process in resonance by degenerating singlet and triplet levels.^10^ Thus we then performed first principles TD‐DFT investigations, natural bond orbital (NBO) analysis, and spin–orbit coupling (SOC) calculations.^29^, ^30^ Ground state electronic structure of T‐CNDF‐T‐*t*Cz shows that HOMO‐2 (*E*_H2_ = −6.305, Figure S1c, Supporting Information), HOMO‐1 (*E*_H1_ = −6.305, Figure S1d, Supporting Information), and HOMO (*E*_H_ = −6.219, Figure S1e, Supporting Information), as well as the LUMO (*E*_L_ = −2.541, Figure S1f, Supporting Information) and LUMO+1 (*E*_L1_ = −2.541, Figure S1g, Supporting Information) are nearly degenerated. Accordingly, from the TD‐DFT calculated excited states of T‐CNDF‐T‐*t*Cz, singlet (S_1_–S_6_) and triplet excited states (T_2_–T_6_) have similar energy levels (Table S3, Supporting Information), which are close to T_1_ with small Δ*E*_SnTn_s (*n* ≤ 6) to support an efficient multichannel RISC process. In contrast, S‐CNDF‐S‐*t*Cz and S‐CNDF‐D‐*t*Cz with fewer donor/acceptor units, the *S*_n_ and *T*_n_ states are considerably different. As illustrated in **Figure** **2** and Table S4 (Supporting Information), the numbers of the valid RISC channels with both small Δ*E*_ST_ (≤0.37 eV) and high SOC values (≥0.3 cm^−1^)^29^, ^30^ are determined to be 0, 5, and 8, respectively, for S‐CNDF‐S‐*t*Cz, S‐CNDF‐D‐*t*Cz, and T‐CNDF‐T‐*t*Cz. All these results suggest that the RISC process from *T*_n_ to *S*_n_ in T‐CNDF‐T‐*t*Cz is more effective than in the reference compounds. Consequently, the multi‐(donor/acceptor) molecule possesses much efficient triplet‐harnessing abilities, and thereby may enhance their light‐emitting performances under the optical or electrical excitations.

**Figure 2:**
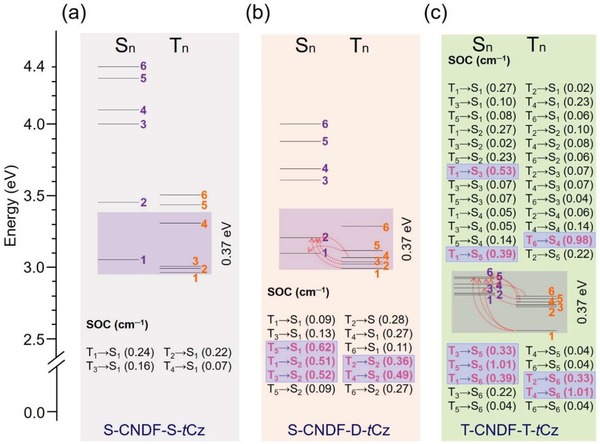
Proposed mechanism for facile RISC channel (both ∆*E*_ST_ ≤ 0.37 eV, and SOC ≥ 0.3 cm^−1^)^29^, ^30^ in multi‐(donor/acceptor)‐based TADF materials. TD‐DFT calculated energy level diagram and the corresponding SOC constants of S‐CNDF‐S‐*t*Cz (a), S‐CNDF‐D‐*t*Cz (b), and T‐CNDF‐T‐*t*Cz (c). The valid RISC channels are highlighted by pink color.

All compounds were synthesized by Pd‐catalyzed Suzuki crossing‐coupling reactions of different halogen (Br or I)‐substituted *t*Cz units with the corresponding CNDF‐borate in moderate yield (Scheme S1, Supporting Information). Their molecular structures were well characterized by ^1^H NMR, ^13^C NMR, high‐resolution mass spectrometry (HRMS), and single crystal structure analysis. As shown in **Figure** **3**, the crystal structures confirm that the minimum distance between nonhydrogen atoms in *t*Cz and CNDF, which are found to be the atoms that directly connect to the benzene bridge, is ≈2.9 Å, which may allow the efficient TSCT to achieve delayed fluorescence. In addition, all the molecular structures are highly twisted, the dihedral angles between the *t*Cz units and the adjacent phenyl bridges are 55.2° for S‐CNDF‐S‐*t*Cz, 61.7° for S‐CNDF‐D‐*t*Cz, and 74.9° for T‐CNDF‐T‐*t*Cz, illustrating the coexistence of TSCT and TBCT effects within these compounds. Notably, the packing diagram of T‐CNDF‐T‐*t*Cz demonstrates that the multi‐(donor/acceptor) molecule exhibits negligible intermolecular interactions (Figure S2, Supporting Information), which makes it a suitable emitter for the nondoped OLEDs. All these results are in close agreement with the conclusions obtained from the TD‐DFT simulations. Therefore, we anticipate that the multi‐(donor/acceptor) molecule combining the TSCT and TBCT effects could be a good candidate for high‐performance TADF‐OLEDs.

**Figure 3:**
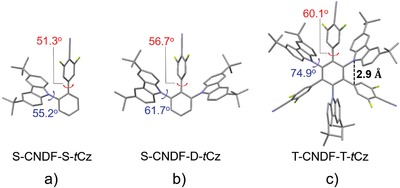
a–c) The single‐crystal structures of S‐CNDF‐S‐*t*Cz (a), S‐CNDFD‐*t*Cz (b), and T‐CNDF‐T‐*t*Cz (c). The values of dihedral angels and distances are the averaged ones obtained from the corresponding crystal structures. For all crystal structures, the disordered solvent molecules and hydrogen atoms are omitted for the sake of clarity. Carbon, gray; nitrogen, blue; fluorine, yellow green.

The UV–vis absorption in chloroform (1 × 10^−4^ m) and photoluminescence (PL) spectra in neat films of S‐CNDF‐S‐*t*Cz, S‐CNDF‐D‐*t*Cz, and T‐CNDF‐T‐*t*Cz are shown in **Figure** **4**, and data are summarized in **Table** **1**. All emitters demonstrate an absorption band at about 330 nm attributed to the local transition of ph‐*t*Cz or ph‐CNDF, and a CT band in the range of 360–420 nm originating from the CT‐transition from the donor to the acceptor. The emission peak of T‐CNDF‐T‐*t*Cz reveals a bathochromic shift of approximately 30 nm compared with S‐CNDF‐S‐*t*Cz or S‐CNDF‐D‐*t*Cz due to the more D–A units. The CT characteristics of these compounds are also inferred by the absorption and emission spectra in solvents with different polarities (Figure S3, Supporting Information). Taking T‐CNDF‐T‐*t*Cz as an example, with increasing solvent polarity, the emission maximum displays a distinct positive solvatochromism (e.g., λ_PLmax_ = 450 nm in toluene, 472 nm in chloroform, and 550 nm in acetone). According to the onsets of fluorescence and phosphorescence spectra of these emitters in their neat films, the Δ*E*_ST_ of S‐CNDF‐S‐*t*Cz, S‐CNDF‐D‐*t*Cz, and T‐CNDF‐T‐*t*Cz are estimated to be 0.24, 0.21, and 0.03 eV, respectively, confirming the multi‐(donor/acceptor) molecule could degenerate frontier orbitals and thus result in a small energy gap, which will effectively improve the RISC process by means of up‐conversion from *T*_n_ to *S*_n_.

**Figure 4:**
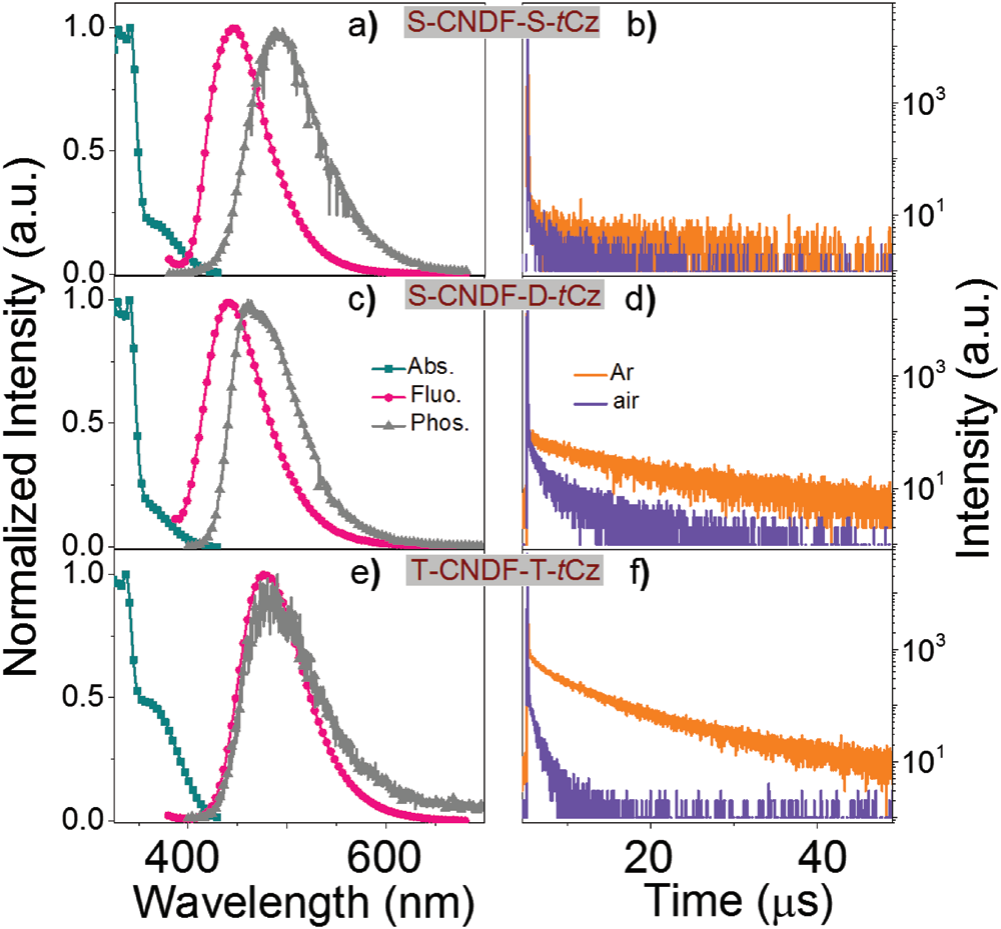
a,c,e) Normalized absorption spectra (green‐blue trace) measured at room temperature in chloroform (1 × 10^−4^ m), normalized fluorescence spectra (red trace) at room temperature, and phosphorescence spectra (gray trace) at 77 K in neat films of S‐CNDF‐S‐*t*Cz (a), S‐CNDF‐D‐*t*Cz (c), and T‐CNDF‐T‐*t*Cz (e). b,d,f) Fluorescence decay curves of S‐CNDF‐S‐*t*Cz (b), S‐CNDF‐D‐*t*Cz (d), and T‐CNDF‐T‐*t*Cz (f) in neat film states at 298 K (monitored at 460 nm) under aerated (purple color) and degassed (orange color) condition.

**Table 1:** Photophysical parameters of S‐CNDF‐S‐*t*Cz, S‐CNDF‐D‐*t*Cz, and T‐CNDF‐T‐*t*Cz

Compound	λ_abs_ [nm]^a)^	λ_em_ [nm]^b)^	*E*_S_ */E*_T_ [eV]^c)^	Δ*E*_ST_ [eV]^d)^	τ_p_ [ns]^e)^	τ_d_ [µs]^f)^	*Φ*_PL_/*Φ*_F_ [%]^g)^
S‐CNDF‐S‐*t*Cz	340/367	476/445	3.08/2.84	0.24	17.0	1.83/54.3	42/31
S‐CNDF‐D‐*t*Cz	340/366	466/441	3.11/2.90	0.21	14.1	2.20/13.4	19/6
T‐CNDF‐T‐*t*Cz	337/362	472/477	2.88/2.85	0.03	21.3	1.65/7.79	76/19

^a)^ Measured in chloroform (1 × 10^−4^ m) at room temperature

^b)^ Measured in chloroform (1 × 10^−4^ m) (former) and neat film (latter) at room temperature, respectively

^c)^ *E*_S_ and *E*_T_ obtained from the onsets of fluorescence and phosphorescence spectra of these emitters in the neat films, respectively

^d)^ Δ*E*_ST_ calculated from *E*_S_−*E*_T_

^e)^ Prompt and

^f)^ Delayed fluorescence lifetimes in the neat films at room temperature, fitted from the time‐resolved decays

^g)^ *Φ*_PL_ and *Φ*_F_ represent the PLQYs of the deoxygenated and air‐saturated neat films, respectively (excitation wavelength is 360 nm).

Subsequently, we conducted the measurement for the transient PL decay curves of these compounds at room temperature. As shown in Figures S4, S5, S6, and Table S1 in the Supporting Information, in degassed chloroform, T‐CNDF‐T‐*t*Cz displays a distinctive delayed emission with a lifetime (τ_d_) of 7.5 µs, together with a prompt emission with a lifetime (τ_p_) of 13 ns. The delayed component percentage is 92% and the prompt component is 8%. However, when exposing this solution to air, the delayed component is inconspicuous, indicating that the delayed emission of T‐CNDF‐T‐*t*Cz is originated from the triplets which are effectively quenched by oxygen (Figure S5e, Supporting Information).^16^ Similar experimental phenomena could also be observed in its nondoped film (Figure 4f). The temperature dependent time‐resolved measurements for S‐CNDF‐S‐*t*Cz, S‐CNDF‐D‐*t*Cz and T‐CNDF‐T‐*t*Cz were performed as shown in **Figure** **5**. All the curves exhibit two clear components, prompt fluorescence (PF) in nanosecond scale and DF in microsecond scale in these three compounds. The positive temperature dependence of the decays in DF region and linear proportionality of DF intensity versus laser pumping power reveal the monomolecular process, confirming their TADF mechanism.

**Figure 5:**
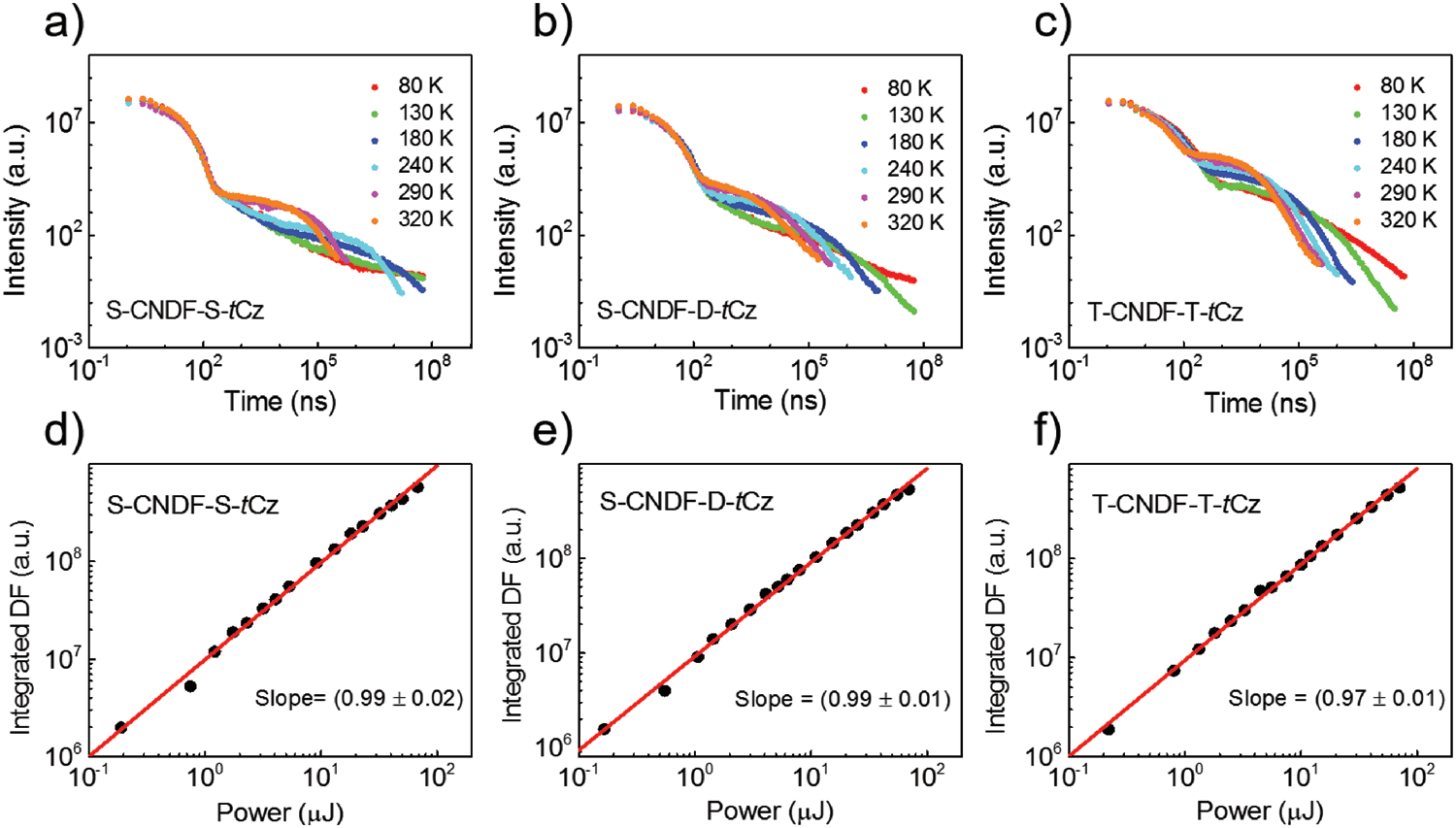
a–c) Temperature‐dependent time‐resolved decays of S‐CNDF‐S‐*t*Cz (a), S‐CNDF‐D‐*t*Cz (b), and T‐CNDF‐T‐*t*Cz (c) in neat films from 320 to 80 K. d–f) Power‐dependent measurements of S‐CNDF‐S‐*t*Cz (d), S‐CNDF‐D‐*t*Cz (e), and T‐CNDF‐T‐*t*Cz (f) with delay/integration time of 0.6/200, 0.6/200, and 0.5/100 µs, respectively, collected at room temperature.

The TD‐DFT calculations forecast that both TBCT and TSCT are involved in the TADF emission in these compounds. To clarify the suggested mechanism, a *para*‐substituted molecule *p*‐S‐CNDF‐S‐*t*Cz was investigated in comparison to its isomer S‐ CNDF‐S‐*t*Cz with the D unit substituted at the *ortho* position of the A unit. In *p*‐S‐CNDF‐S‐*t*Cz, the TSCT seems not to be effective as the *para*‐substitution increases the spatial distance between the D and A units. As shown in Figure S7 in the Supporting Information, a strong CT character is clearly observed, which is safely attributed to the TBCT effect. Thus it is proposed that the TBCT is active and dominant in the *para*‐substituted molecule *p*‐S‐CNDF‐S‐*t*Cz. As expected, no TADF is observed due to the large energy gap (0.44 eV, Figure S8, Supporting Information). This indicates that only TBCT exists in *p*‐S‐CNDF‐S‐*t*Cz to give the CT character. The results give clear evidence that the contribution of TADF in S‐CNDF‐S‐*t*Cz originates from both TBCT and TSCT effects. With the increasing numbers of D/A units in S‐CNDF‐D‐*t*Cz and T‐CNDF‐T‐*t*Cz, a large density of triplet pathways given by more D/A channels via TBCT and TSCT and there is no doubt that TSCT accelerates the RISC process due to the further reduced Δ*E*_ST_ in T‐CNDF‐T‐*t*Cz. Therefore, more efficient TADF is observed in the multi‐(donor/acceptor) system. All these results are in good agreement with the TD‐DFT calculated data.

Besides, the anti‐stacking crystal pattern of T‐CNDF‐T‐*t*Cz may allow it to avoid fluorescence quenching in the pristine state.^27^ Then, we evaluated the PLQYs of these three compounds in the neat films. As listed in Table 1, the absolute PLQYs in air‐saturated thin film are 31% for S‐CNDF‐S‐*t*Cz, 6% for S‐CNDF‐D‐*t*Cz, and 19% for T‐CNDF‐T‐*t*Cz. In contrast, the degassed samples give the corresponding values of 42%, 19% and 76%, sequentially. Obviously, in the presence of oxygen, the PLQY of T‐CNDF‐T‐*t*Cz has been significantly reduced, which may be ascribed to the larger density of T_n_s which are easily quenched by oxygen. The rate constants of RISC (*k*_RISC_) were estimated using a previously reported method^31^ to be (0.12 ± 0.05) × 10^5^, (2.27 ± 0.30) × 10^5^, and (5.07 ± 0.65) × 10^5^ s^−1^, respectively, for S‐CNDF‐S‐*t*Cz, S‐CNDF‐D‐*t*Cz, and T‐CNDF‐T‐*t*Cz (Figure S9, Supporting Information). In comparison with the reference molecules, the *k*_RISC_ value of multi‐(donor/acceptor) molecule was distinctly enhanced, evidencing a more efficient RISC process in T‐CNDF‐T‐*t*Cz due to the increased density of triplet pathways. The promising luminescent behavior of T‐CNDF‐T‐*t*Cz both in solution and film state clearly demonstrated the success of our unique design strategy for efficient TADF features.

We fabricated the solution‐processed devices using the multi‐(donor/acceptor) emitter, T‐CNDF‐T‐*t*Cz, as a nondoped emitting layer, where the used solvent is chlorobenzene. The structure of device A is ITO/PEDOT:PSS (40 nm)/T‐CNDF‐T‐*t*Cz (50 nm)/DPEPO (10 nm)/TmPyPB (50 nm)/Liq (1 nm)/Al (100 nm) (**Figure** **6**a), where poly(3,4‐ethylenedioxythiophene):poly(styrenesulfonic acid) (PEDOT:PSS) and 8‐hydroxyquinolinolato‐lithium (Liq) served as the hole‐ and electron‐injection layer, respectively; 1,3,5‐tri(m‐pyrid‐3‐yl‐phenyl)benzene (TmPyPB) acted as the electron‐transporting layer; and bis(2‐(diphenylphosphino)phenyl)ether oxide (DPEPO) was used as the hole‐blocking layer. We also fabricated two reference devices employing S‐CNDF‐S‐*t*Cz (device B) and S‐CNDF‐D‐*t*Cz (device C) in the same device configurations for comparison.

**Figure 6:**
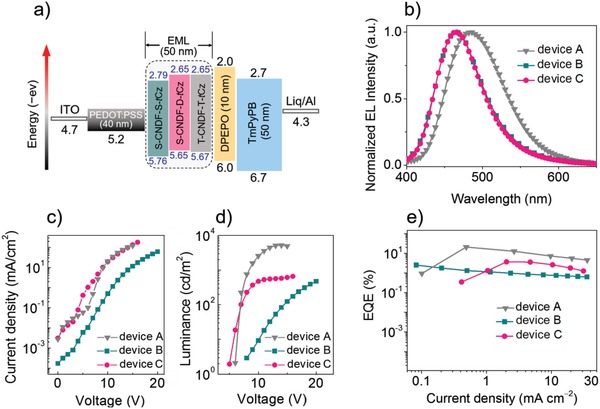
a) Solution‐processed device configurations of the S‐CNDF‐S‐*t*Cz‐, S‐CNDF‐D‐*t*Cz‐, and T‐CNDF‐T‐*t*Cz‐employing devices, A, B, and C. b) EL spectra, c) voltage–current density (*V–J*) characteristics, d) voltage–luminance (*V–L*) characteristics, and e) external quantum efficiency–current density characteristics of the devices A, B, and C.

The voltage–current density, voltage–brightness characteristics, EL spectrum and the efficiency as a function of the current density of these devices are illustrated in Figure 6 and Figures S12 and S13 (Supporting Information) and the data are summarized in **Table** **2**. Devices A and C exhibited similar turn‐on voltages at around 6 V, however, device B (S‐CNDF‐S‐*t*Cz) showed a higher one at about 10 V, which is mainly due to the high LUMO level (−2.65 eV) of S‐CNDF‐S‐*t*Cz and the less ordered molecular packing for charge hopping in the film state. Device A based on T‐CNDF‐T‐*t*Cz shows a sky‐blue emission peaking at 484 nm, a maximum brightness (*B*_max_) of 5210 cd m^−2^, a maximum current efficiency (CE_max_) of 46.4 cd A^−1^, a maximum power efficiency (PE_max_) of 20.8 lm W^−1^, and an EQE_max_ of 21.0%. To the best of our knowledge, the EL performances of the multi‐(donor/acceptor)‐based device are evidently superior to those reported previously, and achieved the record efficiency among sky‐blue OLEDs fabricated by solution process. Comparatively, the EL performances of the reference devices B and C are much inferior, with the *B*_max_, CE_max_, PE_max_, and EQE_max_ of 474 cd m^−2^, 3.6 cd A^−1^, 1.4 lm W^−1^, and 2.6% for S‐CNDF‐S‐*t*Cz, and 659 cd m^−2^, 5.0 cd A^−1^, 2.2 lm W^−1^, and 3.7% for S‐CNDF‐D‐*t*Cz, respectively. Particularly, the EQEs drastically drop from 21% (T‐CNDF‐T‐*t*Cz, three‐D/A) to 2.6% (S‐CNDF‐S‐*t*Cz, single D/A). Furthermore, the η_r_ (exciton utilization efficiency) of these devices was estimated according to the equation: η_ext_ = γ · η_r_ · *Φ*_PL_ · η_out_, where η_out_ represents the light out‐coupling efficiency (for glass substrates, η_out_ ≈ 25−30%), γ is the charge balance factor (for a proper device, γ = 1). Considering the *Φ*_PL_ of 76% for T‐CNDF‐T‐*t*Cz, barely in its own film, the estimated η_r_ of this multi‐(donor/acceptor)‐based device was approaching 100%, indicating that almost all the electrically generated excitons are radiative. The fact that the higher efficiency in device A may be ascribed to the much higher PLQY and faster RISC process induced by the multi‐(donor/acceptor) nature together with the simultaneous TSCT and TBCT effects in T‐CNDF‐T‐*t*Cz compared with the reference molecules, which clearly evidence the effectiveness and flexibility of our design strategy.

**Table 2:** Summary of the EL Data of the devices A, B, and C employing T‐CNDF‐T‐*t*Cz, S‐CNDF‐S‐*t*Cz, and S‐CNDF‐D‐*t*Cz, respectively

Device	Emitting layers	*V*_on_ [V]^a)^	*B*_max_ [cd m^−2^]^b)^	CE_max_ [cd A^−1^]^c)^	PE_max_ [lm W^−1^]^d)^	EQE_max_ [%]^e)^	λ_em_ [nm]^f)^	CIE [*x*,*y*]^g)^
A	T‐CNDF‐T‐*t*Cz	6.3	5210	46.4	20.8	21.0	484	(0.19, 0.35)
B	S‐CNDF‐S‐*t*Cz	10.1	474	3.6	1.4	2.6	466	(0.16, 0.18)
C	S‐CNDF‐D‐*t*Cz	5.7	659	5.0	2.2	3.7	466	(0.16, 0.17)

^a)^ The operation voltage recorded at a brightness of 10 cd m^−2^

^b)^ The maximum brightness (*B*_max_)

^c)^ The maximum current efficiency (CE_max_)

^d)^ The maximum power efficiency (PE_max_)

^e)^ The maximum external quantum efficiency (EQE_max_)

^f)^ The EL peak wavelength and

^g)^ Commission International de I'Eclairage coordinates.

In summary, we proposed a novel and efficient molecular design strategy for the construction of high‐performance TADF materials by introducing multiple alternating donor and acceptor units onto the π bridge (like benzene ring). The co‐existence of through‐space and through‐bond charge transfer ensures the molecule, targeting for blue emission, to possess a very small energy gap and large transition dipole moment concurrently. Additionally, the highly twisted structure of the multi‐(donor/acceptor) systems can not only efficiently inhibit aggressive quenching to obtain a high PLQY, but also potentially degenerate molecular orbitals, leading to the opening of more RISC channels. Eventually, the three‐(donor/acceptor) TADF molecule, T‐CNDF‐T‐*t*Cz, was ensured with a small singlet–triplet gap, high quantum yield, and fast RISC process. Hence, it shows much superior EL efficiencies than the reference molecules with fewer donor/acceptor units, S‐CNDF‐S‐*t*Cz and S‐CNDF‐D‐*t*Cz, e.g., 21% versus 2.6% and 3.7%, respectively, in terms of EQE_max_. It is worth mentioning that the EQE of the device employing T‐CNDF‐T‐*t*Cz champions blue solution‐processed TADF OLEDs with the neat emitters. Evidently, this facile constructive strategy for TADF molecules would greatly extend the design rationales, which will light up the enthusiasm of scientists to develop more promising TADF materials for flat panel displays and lighting applications.

## Conflict of Interest

The authors declare no conflict of interest.

## Supporting information

Supporting Information
