# Supplementary material for: Achieving 21% External Quantum Efficiency for Nondoped Solution‐Processed Sky‐Blue Thermally Activated Delayed Fluorescence OLEDs by Means of Multi‐(Donor/Acceptor) Emitter with Through‐Space/‐Bond Charge Transfer
Source: Adv Sci (Weinh). 2020 Feb 8;7(7):1902087. doi: 10.1002/advs.201902087 (PMC7141015; doi:10.1002/advs.201902087)
Supplement: Supplementary file 1 — Supporting Information [file ADVS-7-1902087-s001.pdf]

Copyright WILEY-VCH Verlag GmbH & Co. KGaA, 69469 Weinheim, Germany, 2020.

ADVANCED SCIENCE Open Access

Supporting Information

for *Adv. Sci.,* DOI: 10.1002/advs.201902087

Achieving 21% External Quantum Efficiency for Nondoped Solution-Processed Sky-Blue Thermally Activated Delayed Fluorescence OLEDs by Means of Multi-(Donor/Acceptor) Emitter with Through-Space/-Bond Charge Transfer

Xujun Zheng, Rongjuan Huang, Cheng Zhong, Guohua Xie, Weimin Ning, Manli Huang, Fan Ni, Fernando B. Dias,* and Chuluo Yang**

Copyright WILEY-VCH Verlag GmbH & Co. KGaA, 69469 Weinheim, Germany, 2020.

Supporting Information

for *Adv. Sci.*, DOI: 10.1002/advs.201902087

Achieving 21% External Quantum Efficiency for Nondoped Solution-Processed Sky-Blue Thermally Activated Delayed Fluorescence OLEDs by Means of Multi-(Donor/Acceptor) Emitter with Through-Space/-Bond Charge Transfer

Xujun Zheng, Rongjuan Huang, Cheng Zhong, Guohua Xie, Weimin Ning, Manli Huang, Fan Ni, Fernando B. Dias,* and Chuluo Yang**

Dr. X. Zheng, Dr. C. Zhong, W. Ning, M. Huang, Prof. G. Xie, Prof. C. Yang,
Renmin Hospital of Wuhan University, Hubei Key Lab on Organic and Polymeric Optoelectronic Materials, Department of Chemistry, Wuhan University, Wuhan 430072, P. R. China.
E-mail: clyang@whu.edu.cn, guohua.xie@whu.edu.cn

Dr. X. Zheng, Dr. F. Ni, Prof. C. Yang,
Shenzhen Key Laboratory of Polymer Science and Technology, College of Materials Science and Engineering, Shenzhen University, Shenzhen 518060, China.
E-mail: clyang@szu.edu.cn

Dr. R. Huang, Prof. F. B. Dias,
Department of Physics, Organic Electroactive Materials Group, Durham University, Durham, United Kingdom.
E-mail: f.m.b.dias@durham.ac.uk

I. Experimental details

1. General information

All reagents and solvents were purchased from commercial sources and used without further purification. Manipulations were performed under normal atmosphere unless specially noted. Nuclear magnetic resonance (NMR) spectra were recorded at ambient temperature using Bruker AVANCE III 400 spectrometers, with working frequencies of 400 and 100 MHz for ^{1}H and ^{13}C, respectively. Chemical shifts are reported in ppm relative to the residual internal non-deuterated solvent signals ($CDCl_3$: δ = 7.26 ppm). High-resolution MS spectra were measured with a Q-TOF Premier ESI mass spectrometer (Micromass, Manchester, UK), or a MALDI TOF/TOF mass spectrometer (Bruker Daltonics, Bremen, Germany). UV-vis absorption spectra were recorded on a Shimadzu UV-2700 spectrophotometer. Photoluminescence (PL) spectra were processed on a Hitachi F-4600 fluorescence spectrophotometer. The absolute photoluminescence quantum yields (PLQYs) of the film samples were determined using a Quantaurus-QY measurement system (C9920-02, Hamamatsu Photonics), the sample was excited at 350 nm. The PL lifetimes were obtained from a single photon counting spectrometer on Edinburgh Instruments (FLS920) with a Picosecond Pulsed UV-LASTER (LASTER377) as the excitation light source. Thermo gravimetric analyses (TGAs), from 34 to 600 °C, were performed on a NETZSCH STA 449C instrument under nitrogen with a heating rate of 10 °C min^{-1}. Cyclic voltammetries (CVs) were measured on a CHI voltammetric analyzer at room temperature with the conventional three-electrode configuration, consisted of a platinum column working electrode, a platinum wire auxiliary electrode, and an Ag wire pseudo reference electrode. Cyclic voltammograms (CV) were recorded using tetrabutylammonium hexafluorophosphate ($TBAPF_6$) (0.1 M) dissolved in dichloromethane as the supporting electrolyte, and ferrocenium-ferrocene (Fc^+/Fc) as the external standard, at the scan rate of 100 mV s^{-1}. The onset potentials were calculated out from the intersection of two tangents of the rising and background current in cyclic voltammograms.

Prompt fluorescence (PF), delayed fluorescence (DF) and phosphorescence spectra and time-resolved decays were recorded using nanosecond gated luminescence and lifetime measurements (from 800 ps to 1 s) with a high energy pulsed Nd:YAG laser emitting at 355 nm (EKSPLA). Emission was focused onto a spectrograph equipped with 300 lines/mm

grating of 500 nm or 1000 nm based wavelength and detected using a sensitive gated iCCD camera (Stanford Computer Optics) with sub-nanosecond resolution.

2. Computational details

Theoretical simulations were performed using the Gaussian 09 program packages. The geometries were optimized at the B3LYP/6-31G(d) level. And the electronic properties of the emitter were calculated by TD-DFT procedure employing range-separated exchange density functionals at the LC-PBE/6-31G(d) level. The geometries and FMO distributions were visualized using Gaussview.

3. OLED fabrication and measurements

The patterned indium tin-oxide (ITO)-coated glasses were used as the substrates. Before device fabrication, the substrates were cleaned sequentially using acetone and ethanol and then treated in an ultrasonic bath for 10 min respectively in each solvent. After dried with a N_2 gun, the substrates were treated with a UV–ozone surface processor (PL16 series, SenLights Corporation). Then PEDOT:PSS was spin-coated onto the ITO substrate as the hole-injecting layer, which was annealed at 120 ^oC for 10 min. The emissive layers were prepared by spin-coating onto the hole-transporting layer and then annealed at 50^oC for 10 min. The electron-transporting and the cathode materials were thermally evaporated onto the emitter layer in a vacuum chamber. Before taken out of the glove-box, the devices were encapsulated with UV-curable epoxy. The voltage-current-luminance characteristics and the EL spectra were simultaneously measured with PR735 SpectraScan Spectroradiometer and Keithley 2400 sourcemeter unit under ambient atmosphere at room temperature.

4. X-Ray Structural Analysis

Single crystal **S-CNDF-S-*t*Cz**, **S-CNDF-D-*t*Cz**, and **T-CNDF-T-*t*Cz** were achieved from slow evaporation of *ethyl acetate*/*ethanol* solution at room temperature. Single-crystal X-ray-diffraction data were obtained from a Bruker APEX Duo diffractometer through using MoKα radiation (λ = 0.71073 Å) with a□$\omega/2\theta$ scan mode at the temperature of 298 or 77 K. Structure of the crystal was solved by direct methods using the SHELXS-97 software. None-hydrogen atoms were refined anisotropically by full-matrix least-squares calculations on *F*2

using SHELXL-97, while the hydrogen atoms were directly introduced at calculated position and refined in the riding mode. Drawings were produced using Ortep 3 and Mercury 3.3. CCDC-1906685 (**S-CNDF-S-*t*Cz**), CCDC-1906686 (**S-CNDF-D-*t*Cz**), and CCDC-1906688 (**T-CNDF-T-*t*Cz**) contain supplementary crystallographic data. The data can be obtained free of charge from the Cambridge Crystallographic Data Centre via www.ccdc.cam.ac.uk/data_request/cif.

5. Synthetic procedures and characterization data

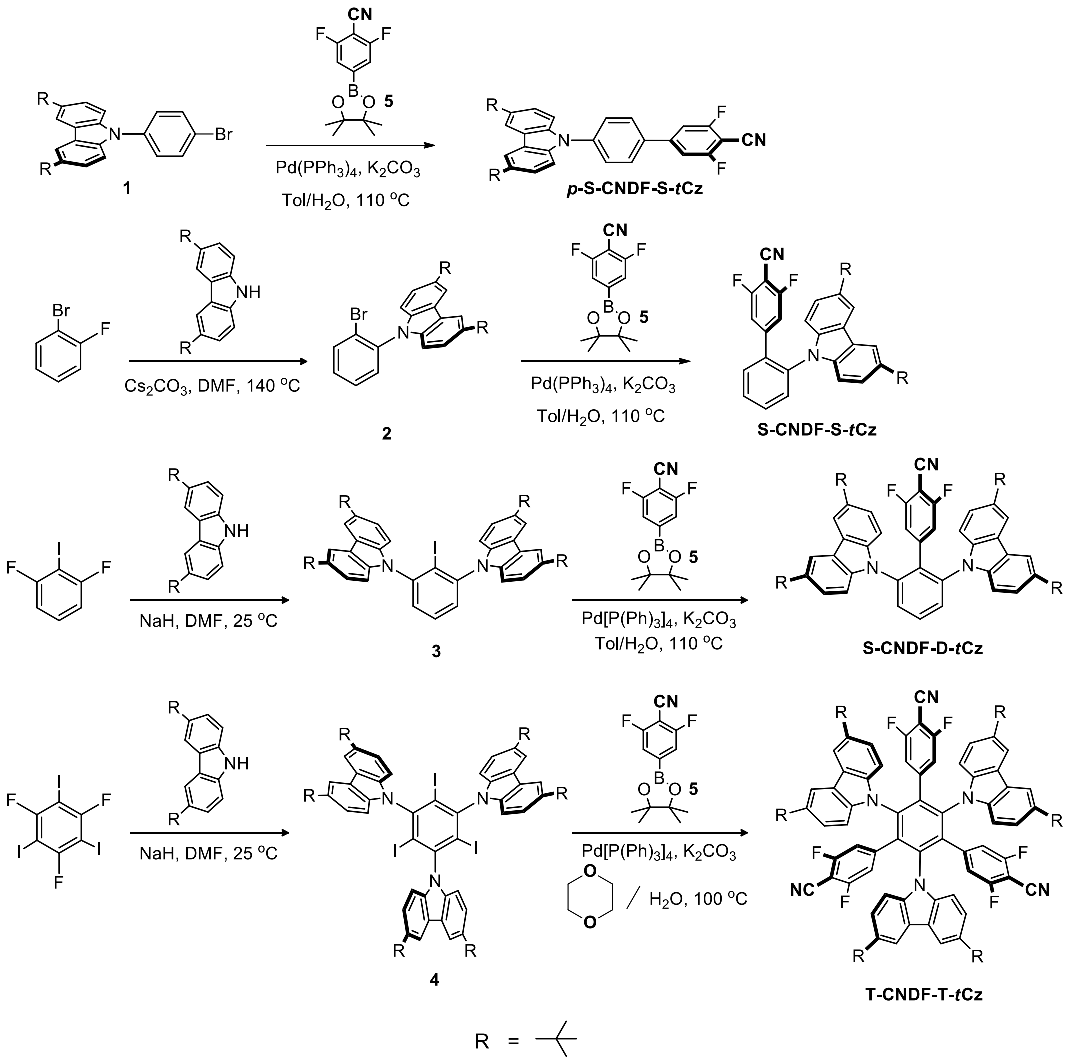

Scheme S1. Synthetic routes to ***p*-S-CNDF-S-*t*Cz**, **S-CNDF-S-*t*Cz**, **S-CNDF-D-*t*Cz**, and **T-CNDF-T-*t*Cz**.

9-(2-bromophenyl)-3,6-di-*tert*-butyl-9*H*-carbazole (2)

A flask was charged with a mixture of 3,6-di-*tert*-butyl-9*H*-carbazole (335 mg, 1.2 mmol), 2-Bromofluorobenzene (173 mg, 1.0 mmol), Cs_2CO_3 (650 mg, 2.0 mmol), and DMF (20 mL). The reaction mixture was stirred for 24 h under argon at 140 °C. After cooled down to room temperature, the mixture was diluted with CH_2Cl_2 (20 mL × 3) and washed with brine, dried under anhydrous Na_2SO_4, then the solvent was removed in vacuum. The crude product was purified by column chromatography (eluent: petroleum ether) to render 0.37 g white solid. Yield: 85.5%. ^{1}H NMR (400 MHz, Chloroform-*d*) δ 8.14 (dd, *J* = 2.0, 0.4 Hz, 2H), 7.85 (dd, *J* = 8.0, 1.6 Hz, 1H), 7.49 (ddd, *J* = 8.0, 7.2, 1.6 Hz, 1H), 7.43 (dt, *J* = 8.0, 2.0 Hz, 3H), 7.39 (ddd, *J* = 8.0, 7.2, 2.0 Hz, 1H), 6.99 (dd, *J* = 8.4, 0.4 Hz, 2H), 1.46 (s, 18H). ^{13}C NMR (100 MHz, Chloroform-*d*) δ 142.8, 139.4, 137.3, 134.1, 131.0, 129.8, 128.7, 123.6, 123.2, 116.3, 109.5, 34.8, 32.0. HRMS (ESI) m/z calcd for $C_{26}H_{29}BrN^+$ $(M + H)^+$ 434.1478, found 434.1479.

9,9'-(2-iodo-1,3-phenylene)bis(3,6-di-*tert*-butyl-9*H*-carbazole) (3)

3,6-di-*tert*-butyl-9*H*-carbazole (670 mg, 2.4 mmol) and sodium hydride (60%, 96 mg, 2.4 mmol) were dissolved in dry DMF (5 mL) under argon condition. After stirring at 25 °C for 0.5 h, 1,3-difluoro-2-iodobenzene (240 mg, 1 mmol) in dry DMF (5 mL) was slowly added, and then the mixture was stirred at this temperature for 12 h. The reaction mixture was poured into cold water and extracted with CH_2Cl_2 (20 mL × 3). After evaporation of the solvent, the residue was purified by column chromatography (eluent: petroleum ether) to give 690 mg white powder. Yield: 90.9%. ^{1}H NMR (400 MHz, Chloroform-*d*) δ 8.17 (d, *J* = 2.0 Hz, 4H), 7.70 (dd, *J* = 8.4, 7.2 Hz, 1H), 7.54 (d, *J* = 7.6 Hz, 2H), 7.51 (dd, *J* = 8.4, 2.0 Hz, 4H), 7.09 (d, *J* = 8.4 Hz, 4H), 1.47 (s, 36H). ^{13}C NMR (100 MHz, Chloroform-*d*) δ 143.5, 143.0, 139.1, 130.6, 130.4, 123.8, 123.3, 116.5, 109.5, 34.8, 32.0. MALDI-TOF m/z calcd for $C_{46}H_{52}IN_2^+$ $(M + H)^+$ 759.3175, found 759.4809.

9,9',9''-(2,4,6-triiodobenzene-1,3,5-triyl)tris(3,6-di-*tert*-butyl-9*H*-carbazole) (4)

3,6-di-tert-butyl-9H-carbazole (1.0 g, 3.6 mmol) and sodium hydride (60%, 144 mg, 3.6 mmol) were dissolved in dry DMF (10 mL) under argon condition. After stirring at 25 °C for 0.5 h, 1,3,5-trifluoro-2,4,6-triiodobenzene (510 mg, 1 mmol) in dry DMF (10 mL) was slowly added, and then the mixture was stirred at this temperature for 12 h. The reaction

mixture was poured into cold water and extracted with CH_2Cl_2 (20 mL × 3). After filtration and evaporation of the solvent, the residue was purified by column chromatography (eluent: petroleum ether) to give 1.15 g white powder. Yield: 89.3%. ^{1}H NMR (400 MHz, Chloroform-*d*) δ 8.19 – 8.11 (dd, *J* = 2.0, 0.4 Hz, 6H), 7.56 (dd, *J* = 8.4, 2.0 Hz, 6H), 7.07 (dd, *J* = 8.4, 0.4 Hz, 6H), 1.46 (s, 54H). ^{13}C NMR (100 MHz, Chloroform-*d*) δ 146.3, 143.5, 136.8, 124.29, 123.4, 116.8, 108.8, 107.2, 34.8, 32.0. HRMS (ESI) m/z calcd for $C_{66}H_{73}I_3N_3^+$ $(M + H)^+$ 1288.2933, found 1288.2947.

4'-(3,6-di-tert-butyl-9H-carbazol-9-yl)-3,5-difluoro-[1,1'-biphenyl]-4-carbonitrile (*p*-S-CNDF-S-*t*Cz)

A flask was charged with **5** (200 mg, 0.75 mmol), **1** (217 mg, 0.5 mmol), $Pd(PPh_3)_4$ (57.6 g, 0.05 mmol), K_2CO_3 (207 mg, 1.5 mmol), H_2O (1 mL) and toluene (3 mL). The reaction mixture was stirred at 110 °C for 24 h under argon. After cooled down to room temperature, the mixture was poured into 20 mL water, extracted with 20 mL × 3 of CH_2Cl_2, washed with brine, and dried over anhydrous Na_2SO_4. After removal of solvent, the crude product was purified by column chromatography over silica using petroleum ether/ ethyl acetate (15/1, v/v) as eluent, followed by recrystallization from CH_2Cl_2/ CH_3OH for three times to yield the pure product as white solid (156 mg). Yield: 63.3 %. ^{1}H NMR (400 MHz, Chloroform-*d*) δ 8.16 (d, *J* = 2.0 Hz, 2H), 7.78 (d, *J* = 8.4 Hz, 2H), 7.75 – 7.69 (m, 2H), 7.49 (dt, *J* = 8.4, 2.0 Hz, 2H), 7.42 (dd, *J* = 8.8, 1.2 Hz, 2H), 7.40 – 7.33 (m, 2H), 1.48 (s, 18H). ^{13}C NMR (100 MHz, Chloroform-*d*) δ 163.5 (dd, *J* = 260, 5 Hz), 148.4 (t, *J* = 10 Hz), 143.5, 140.0, 138.7, 135.1, 128.5, 127.1, 123.8, 123.8, 116.5, 110.5 (dd, *J* = 20, 4 Hz), 109.4, 109.1, 34.8, 32.0.

2'-(3,6-di-*tert*-butyl-9*H*-carbazol-9-yl)-3,5-difluoro-[1,1'-biphenyl]-4-carbonitrile (S-CNDF-S-*t*Cz)

A flask was charged with **4** (199 mg, 0.75 mmol), **1** (217 mg, 0.5 mmol), $Pd(PPh_3)_4$ (57.6 g, 0.05 mmol), K_2CO_3 (207 mg, 1.5 mmol), H_2O (1 mL) and toluene (3 mL). The reaction mixture was stirred at 110 °C for 24 h under argon. After cooled down to room temperature, the mixture was poured into 20 mL water, extracted with 20 mL × 3 of CH_2Cl_2, washed with brine, and dried over anhydrous Na_2SO_4. After removal of solvent, the crude product was purified by column chromatography over silica using petroleum ether/ ethyl acetate (15/1, v/v) as eluent, followed by recrystallization from CH_2Cl_2/ CH_3OH for three times to yield the pure product as white solid (180 mg). Yield: 73.0 %. ^{1}H NMR (400 MHz, Chloroform-*d*)

δ 8.07 (d, *J* = 2.0 Hz, 2H), 7.67 – 7.57 (m, 3H), 7.55 – 7.49 (m, 1H), 7.34 (dd, *J* = 8.6, 2.0 Hz, 2H), 6.92 – 6.87 (m, 2H), 6.77 – 6.70 (m, 2H), 1.43 (s, 18H). ^{13}C NMR (100 MHz, Chloroform-*d*) δ 162.6 (dd, *J* = 260, 5 Hz), 147.2 (t, *J* = 10 Hz), 143.2, 139.4, 135.6, 131.0, 130.8, 130.3, 129.0, 123.8, 123.4, 116.5, 111.7 (dd, *J* = 20, 4 Hz), 109.1, 108.8, 34.7, 32.0. HRMS (ESI) m/z calcd for $C_{33}H_{31}F_2N_2^+$ $(M + H)^+$ 493.2450, found 493.2448.

2',6'-bis(3,6-di-tert-butyl-9H-carbazol-9-yl)-3,5-difluoro-[1,1'-biphenyl]-4-carbonitrile (S-CNDF-D-*t*Cz)

A flask was charged with **4** (398 mg, 1.5 mmol), **2** (380 mg, 0.50 mmol), $Pd(PPh_3)_4$ (57.6 g, 0.05 mmol), K_2CO_3 (414 mg, 3.0 mmol), H_2O (1 mL) and toluene (3 mL). The reaction mixture was stirred at 110 °C for 24 h under argon. After cooled down to room temperature, the mixture was poured into 20 mL water, extracted with 20 mL × 3 of CH_2Cl_2, washed with brine, and dried over anhydrous Na_2SO_4. After removal of solvent, the crude product was purified by column chromatography over silica using petroleum ether/ ethyl acetate (15/1, v/v) as eluent, followed by recrystallization from CH_2Cl_2/ CH_3OH for three times to yield the pure product as white solid (224 mg). Yield: 58.2%. ^{1}H NMR (400 MHz, Chloroform-*d*) δ 8.04 (d, *J* = 1.8 Hz, 4H), 7.83 (dd, *J* = 8.4, 7.2 Hz, 1H), 7.71 (d, *J* = 8.0 Hz, 2H), 7.38 (dd, *J* = 8.4, 1.8 Hz, 4H), 6.97 (d, *J* = 8.4 Hz, 4H), 6.30 – 6.24 (m, 2H), 1.42 (s, 36H). ^{13}C NMR (100 MHz, Chloroform-*d*) δ 161.6 (dd, *J* = 260, 5 Hz), 143.4, 142.7 (t, *J* = 10 Hz), 139.7, 137.8, 137.6, 131.7, 130.6, 123.8, 123.3, 116.5, 112.0 (dd, *J* = 20, 4 Hz), 111.9, 108.8, 108.7, 34.7, 31.9. MALDI-TOF m/z calcd for $C_{53}H_{54}F_2N_3^+$ $(M + H)^+$ 770.4280, found 770.6474.

(r)-5'-(4-cyano-3,5-difluorophenyl)-2',4',6'-tris(3,6-di-tert-butyl-9*H*-carbazol-9-yl)-3,3'',5,5''-tetrafluoro-[1,1':3',1''-terphenyl]-4,4''-dicarbonitrile (T-CNDF-T-*t*Cz)

A flask was charged with **4** (1.06 g, 4 mmol), **3** (644 mg, 0.5 mmol), $Pd(PPh_3)_4$ (57.6 g, 0.05 mmol), K_2CO_3 (552 mg, 4 mmol), H_2O (2 mL) and 1,4-dioxane (6 mL). The reaction mixture was stirred at 100 °C for 24 h under argon. After cooled down to room temperature, the mixture was poured into 20 mL water, extracted with 20 mL × 3 of CH_2Cl_2, washed with brine, and dried over anhydrous Na_2SO_4. After removal of solvent, the crude product was purified by column chromatography over silica using petroleum ether/ ethyl acetate (10/1, v/v) as eluent, followed by recrystallization from CH_2Cl_2/ CH_3OH for three times to yield the pure product as light green solid (247 mg). Yield: 37.4 %. ^{1}H NMR (400 MHz, Chloroform-*d*) δ 7.88 (d, *J* = 1.6 Hz, 6H), 7.42 (dd, *J* = 8.4, 2.0 Hz, 6H), 6.98 – 6.87 (d, *J* =

8.4 Hz, 6H), 6.32 – 6.21 (m, 6H), 1.39 (s, 54H). ^{13}C NMR (100 MHz, Chloroform-*d*) δ 161.3 (dd, *J* = 260, 5 Hz), 144.6, 142.7, 140.2 (t, *J* = 10 Hz), 139.5, 138.1, 124.2, 123.6, 117.1, 112.0, 111.8 (dd, *J* = 20, 4 Hz), 108.4, 108.2, 91.6 (t, *J* = 20 Hz), 34.8, 31.8. MALDI-TOF m/z calcd for $C_{87}H_{79}F_6N_6^+$ $(M + H)^+$ 1321.6270, found 1321.3884.

II. Photophysical, electrochemical, and devices properties

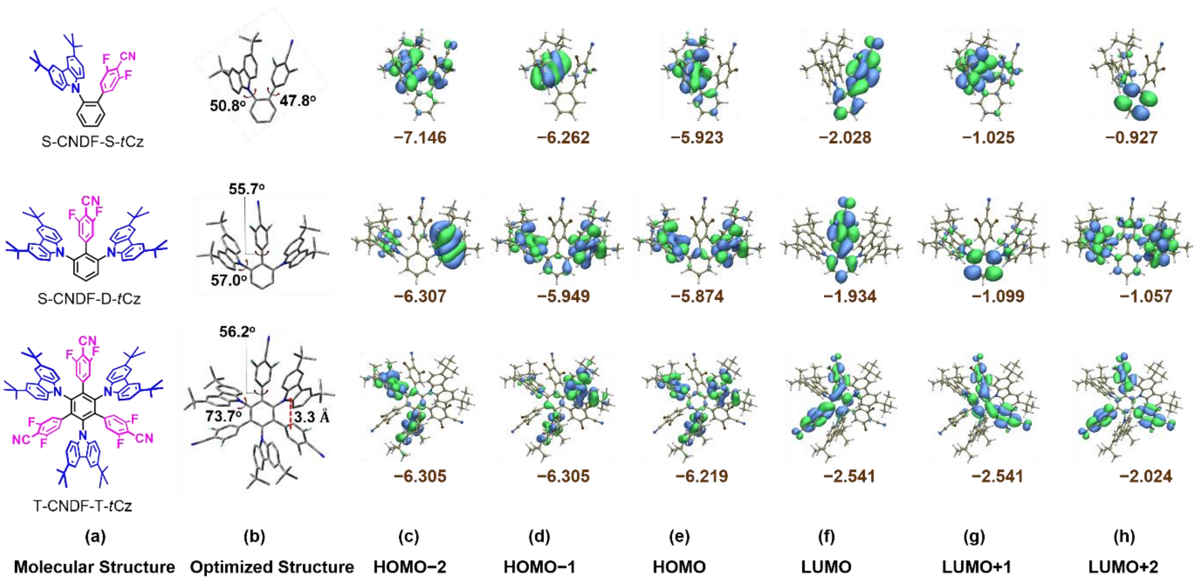

Figure S1. The molecular structures (a), optimized structures (b), HOMO−2 (c)/HOMO−1 (d)/HOMO (e)/LUMO (f)/LUMO+1 (g)/LUMO+2 (h) distributions, and the corresponding energy levels of **S-CNDF-S-*t*Cz**, **S-CNDF-D-*t*Cz**, and **T-CNDF-T-*t*Cz**.

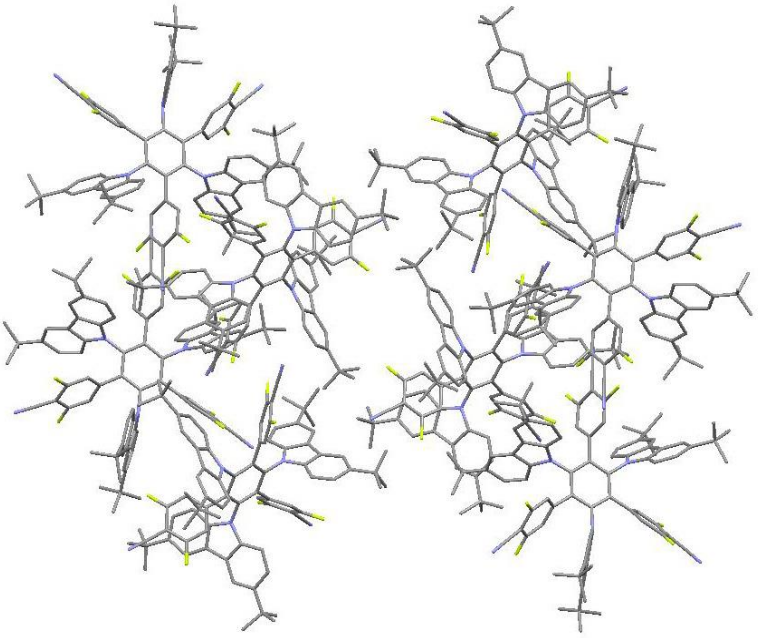

Figure S2. The packing diagrams of **T-CNDF-T-*t*Cz**. The disordered solvent molecules and hydrogen atoms are omitted for the sake of clarity. Carbon, gray; nitrogen, blue; fluorine, yellow green.

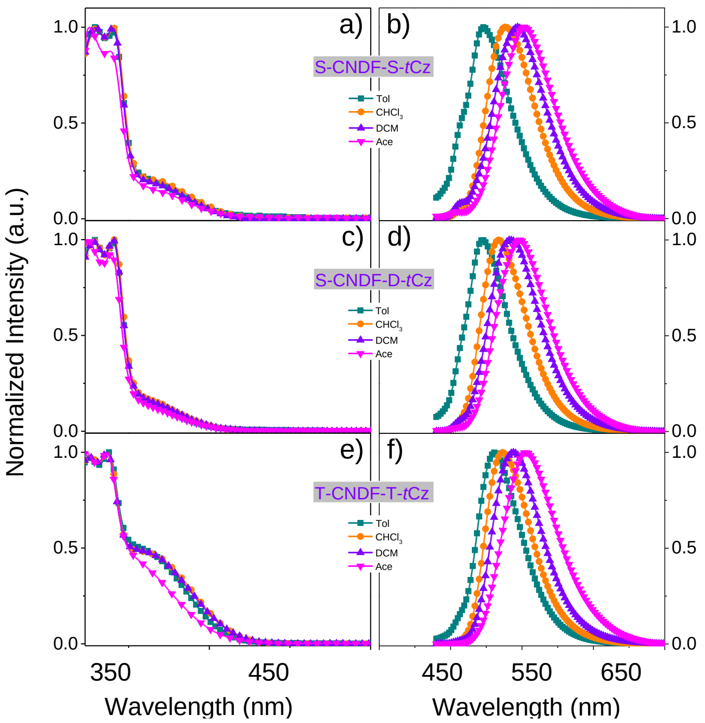

Figure S3. Normalized absorption and fluorescence spectra of **S-CNDF-S-*t*Cz** (a and b), **S-CNDF-D-*t*Cz** (c and d), and **T-CNDF-T-*t*Cz** (e and f) in solvents with different polarities under an excitation of 360 nm. Here, Tol denotes toluene; $CHCl_3$ denotes chloroform; DCM denotes dichloromethane; and ACE denotes acetone.

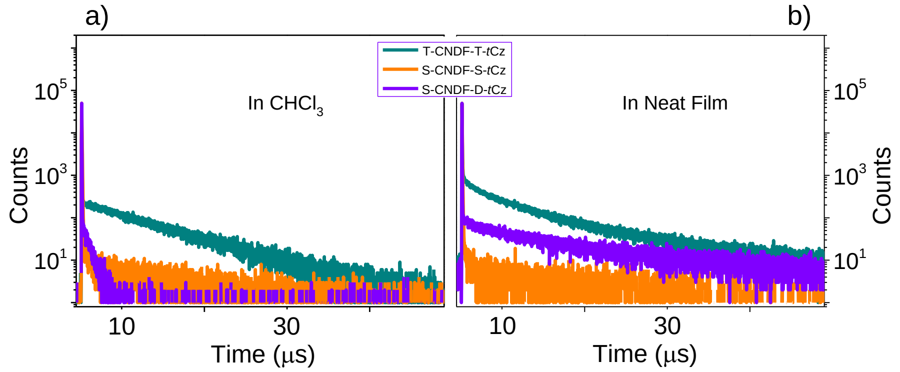

Figure S4. Fluorescence decay curves of **S-CNDF-S-*t*Cz** (orange trace), **S-CNDF-D-*t*Cz** (purple trace), and **T-CNDF-T-*t*Cz** (green blue trace) in N_2-saturated $CHCl_3$ solution (Concentration: 1×10^{-4} M) and in N_2-saturated neat film 298 K (monitored at 460 nm). Excitation wavelength is 377 nm.

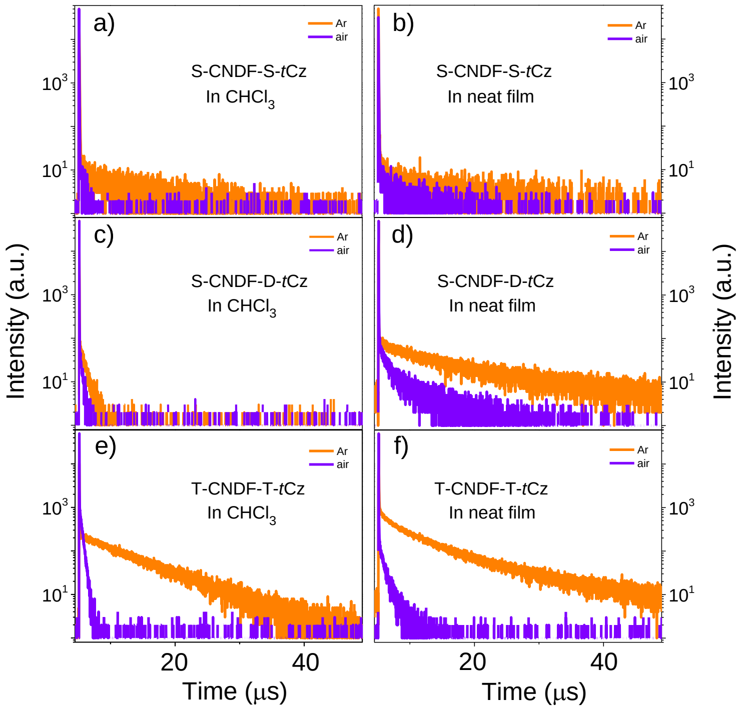

Figure S5. Fluorescence decay curves of **S-CNDF-S-*t*Cz**, **S-CNDF-D-*t*Cz**, and **T-CNDF-T-*t*Cz** in $CHCl_3$ solution (left) and in neat film (right) at 298 K (monitored at 460 nm) under aerated (purple color) and degassed (orange color) condition. Concentration: 1×10^{-4} M; Excitation wavelength is 377 nm.

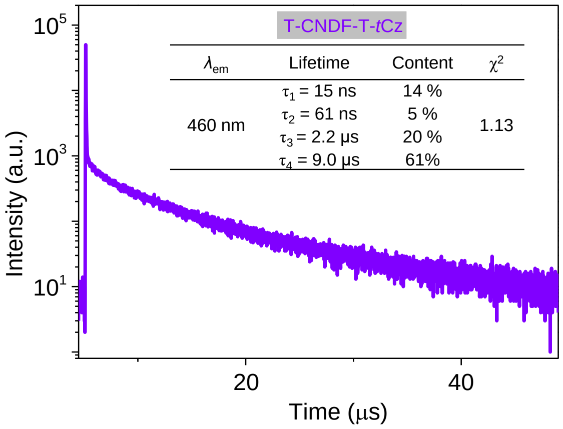

Figure S6. Fluorescence decay curve of **T-CNDF-T-*t*Cz** in N_2-saturated neat film at 298 K (monitored at 460 nm). Excitation wavelength is 377 nm. Inset: the fitted lifetime data of **T-CNDF-T-*t*Cz**.

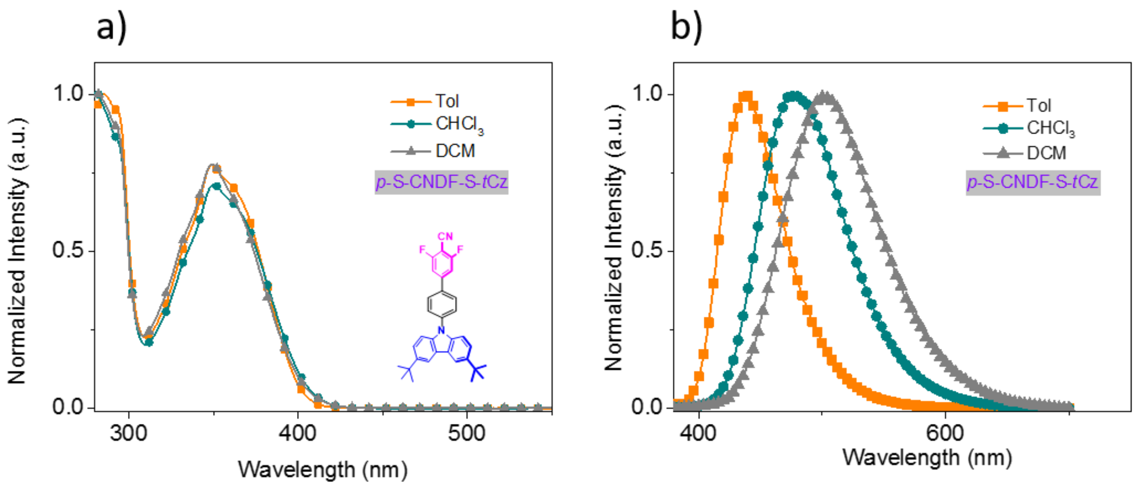

Figure S7. a) UV-Vis absorption and b) fluorescence spectra of ***p*-S-CNDF-S-*t*Cz** in solvents with different polarities under an excitation of 360 nm. Inset shows the molecular structure of ***p*-S-CNDF-S-*t*Cz**. Here, Tol denotes toluene; $CHCl_3$ denotes chloroform; and DCM denotes dichloromethane.

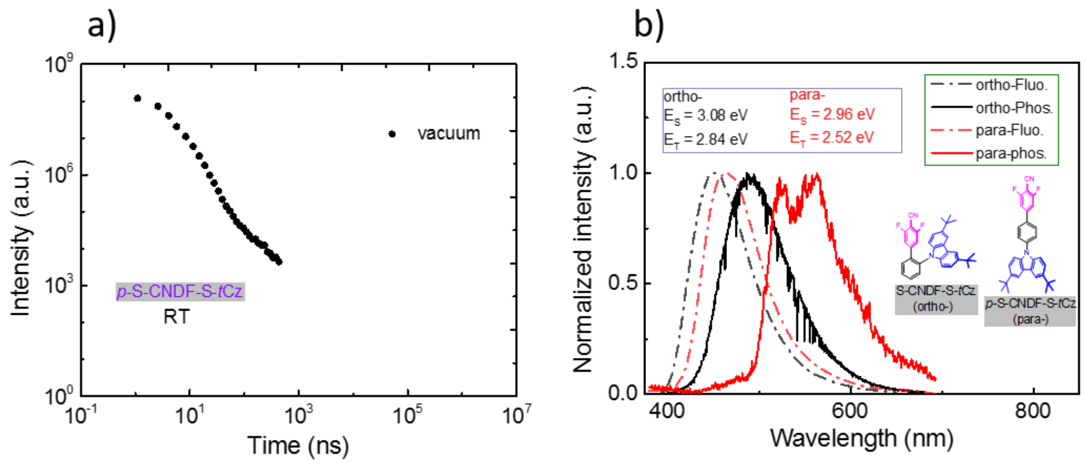

Figure S8. a) Time-resolved decays of ***p*-S-CNDF-S-*t*Cz** in neat films at 298 K under vacuum; b) The comparison of the fluorescence (dotted line) and phosphorescence spectra (solid line) of **S-CNDF-S-*t*Cz** and ***p*-S-CNDF-S-*t*Cz** in neat films.

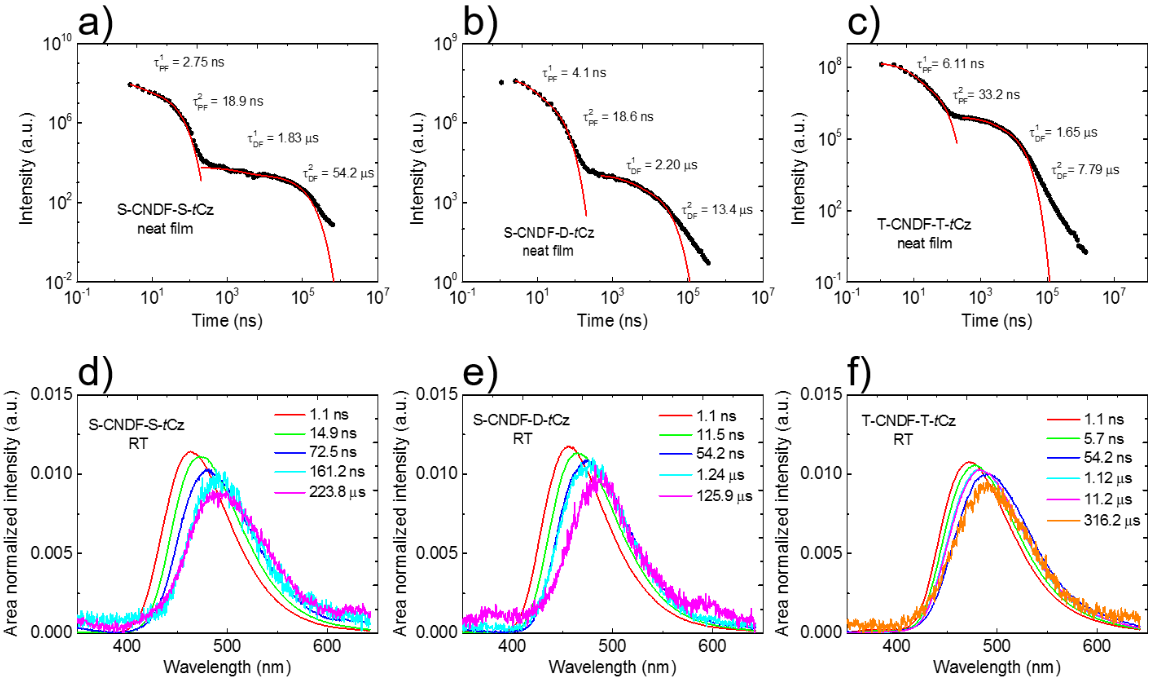

Figure S9. Time-resolved decays (top) and spectra (bottom) of **S-CNDF-S-*t*Cz** (a and d), **S-CNDF-D-*t*Cz** (b and e), and **T-CNDF-T-*t*Cz** (c and f) in neat films at 298 K, and the corresponding fitting lifetimes (a, b, and c), respectively.

Table S1. The fitted photoluminescence lifetime data of **S-CNDF-S-*t*Cz**, **S-CNDF-D-*t*Cz**, and **T-CNDF-T-*t*Cz** in N_2-saturated $CHCl_3$ (Concentration: 1×10^{-4} M.), and in neat film at 298 K.

States	Compounds	λ_{em}	Lifetime	Content	χ^2
In $CHCl_3$	S-CNDF-S-*t*Cz	460 nm	τ_1 = 30 ns<br>τ_2 = 1.6 μs	99 %<br>1 %	1.23
	S-CNDF-D-*t*Cz	460 nm	τ_1 = 14 ns<br>τ_2 = 0.7 μs	96 %<br>4 %	1.05
	T-CNDF-T-*t*Cz	460 nm	τ_1 = 13 ns<br>τ_2 = 7.5 μs	8 %<br>92 %	1.01
In neat film	S-CNDF-S-*t*Cz	460 nm	τ_1 = 12 ns<br>τ_2 = 27 ns	35 %<br>65 %	1.46
	S-CNDF-D-*t*Cz	460 nm	τ_1 = 10 ns<br>τ_2 = 9.4 μs	55 %<br>45 %	1.21
	T-CNDF-T-*t*Cz	460 nm	τ_1 = 15 ns<br>τ_2 = 61 ns<br>τ_3 = 2.2 μs<br>τ_4 = 9.0 μs	14 %<br>5 %<br>20 %<br>61 %	1.13

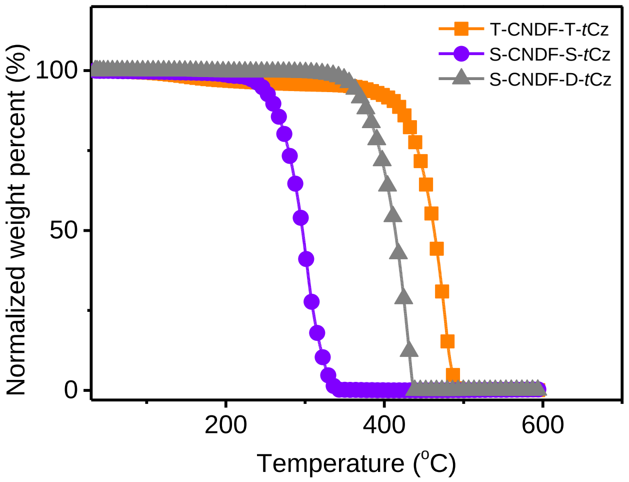

Figure S10. TGA thermogram of **S-CNDF-S-*t*Cz**, **S-CNDF-D-*t*Cz**, and **T-CNDF-T-*t*Cz**.

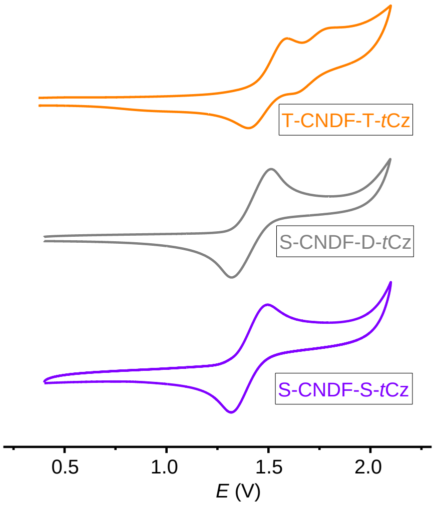

Figure S11. Cyclic voltammogram of **S-CNDF-S-*t*Cz**, **S-CNDF-D-*t*Cz**, and **T-CNDF-T-*t*Cz**. The oxidation potential was determined relative to Ag/Ag^+ in 1×10^{-3} $mol \cdot L^{-1}$ CH_2Cl_2 solution, using Fc/Fc^+ as the internal reference.

Table S2. Physical Properties of S-CNDF-S-*t*Cz, S-CNDF-D-*t*Cz, and T-CNDF-T-*t*Cz.

Compound	λ_{abs}(nm)[a]	λ_{em}(nm)[b]	T_g(°C)[c]	HOMO (eV)	LUMO (eV)	E_g (eV)[d]
**S-CNDF-S-*t*Cz**	340/367	476/445	246	-5.65	-2.65	3.00
**S-CNDF-D-*t*Cz**	340/366	466/441	363	-5.67	-2.65	3.02
**T-CNDF-T-*t*Cz**	337/362	472/477	390	-5.76	-2.79	2.97

[a]Measured in chloroform (1×10^{-4} M) at room temperature. [b]Measured in chloroform (1×10^{-4} M) (former) and non-doped film (latter) at room temperature, respectively. [c]Measured by TGA. [d]E_g obtained from the intersection of the normalized absorption spectra. HOMO determined by the onset potential of the oxidation curves; LUMO levels were calculated by using HOMO levels and E_g.

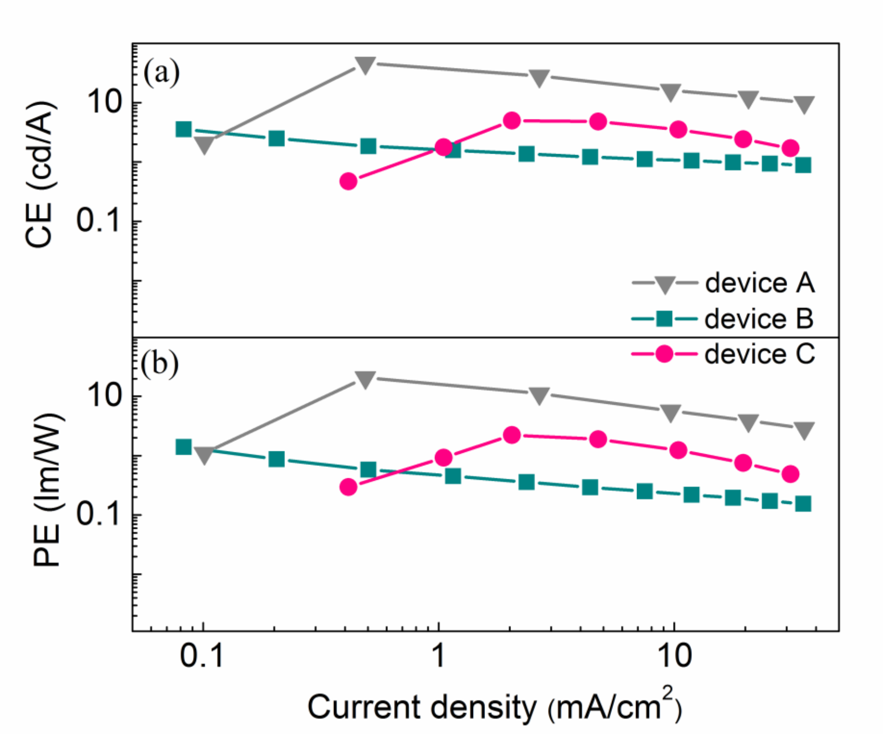

Figure S12. Current efficiency (CE) and power efficiency (PE) versus current density curves for the devices A, B, and C.

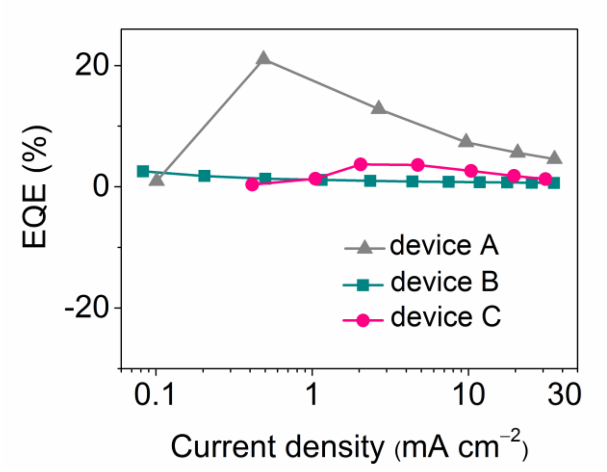

Figure S13. External quantum efficiency (EQE) versus current density curves for the devices A, B, and C.

Table S3. The TD-DFT calculated energy level, and transition configurations of singlet (S_1─S_6) and triplet (T_1─T_6) excited states of S-CNDF-S-*t*Cz, S-CNDF-D-*t*Cz, and T-CNDF-T-*t*Cz.

Compound	S_n/T_n	eV-S	nm-S	f-S	D_idx-S	Sr-S	t_idx-S	orb-S	eV-T	D_idx-T	Sr-T	t_idx-T	orb-T
S-CNDF-S-*t*Cz	1	3.0781	402.79	0.008	3.43	0.2222	1.71	H-L:0.99	2.9496	1.227	0.7182	-0.79	H-L:0.255;H1-L1:0.199
	2	3.4765	356.63	0.003	3.666	0.2133	1.889	H1-L:0.993	2.9835	0.165	0.7784	-1.807	H3-L:0.267;H1-L1:0.234
	3	4.0306	307.61	0.05	0.346	0.7058	-1.056	H-L1:0.908;H1-L5:0.044	2.9889	2.501	0.5217	0.68	H-L:0.625;H1-L1:0.137
	4	4.1258	300.51	0.03	3.772	0.376	1.954	H-L2:0.945	3.33	0.395	0.5959	-0.927	H-L1:0.73;H2-L5:0.039
	5	4.3482	285.14	0.015	3.101	0.414	1.347	H-L3:0.684;H2-L:0.268	3.4032	2.568	0.5616	0.618	H1-L:0.597;H5-L2:0.077
	6	4.4136	280.91	0.024	3.009	0.5463	1.137	H2-L:0.632;H-L3:0.236	3.5145	1.685	0.6387	-0.382	H1-L:0.226;H-L2:0.166
S-CNDF-D-*t*Cz	1	3.1333	395.7	4E-04	0.913	0.2639	-1.152	H-L:0.979	2.9862	0.251	0.5287	-1.55	H-L:0.298;H2-L2:0.19
	2	3.2206	384.97	0.018	1.053	0.2881	-1.067	H1-L:0.976	3.0016	0.669	0.6258	-2.255	H-L:0.161;H3-L2:0.156
	3	3.6256	341.97	0.006	3.385	0.2303	1.766	H2-L:0.967;H3-L:0.023	3.0184	0.288	0.5922	-2.053	H-L:0.204;H6-L:0.163
	4	3.6704	337.8	0.005	3.653	0.1956	2.027	H3-L:0.967;H2-L:0.023	3.0415	0.8	0.7078	-1.323	H6-L:0.211;H4-L:0.161
	5	3.8921	318.55	0.08	2.305	0.3068	0.889	H-L1:0.909;H-L2:0.069	3.1037	0.551	0.4875	-1.532	H1-L:0.64;H-L2:0.086
	6	4.0054	309.54	0.07	0.048	0.6408	-3.46	H-L2:0.514;H1-L3:0.261	3.3002	0.046	0.5925	-2.119	H-L3:0.383;H1-L2:0.345
T-CNDF-T-*t*Cz	1	2.9225	424.25	0.008	1.258	0.2512	-1.668	H-L:0.927	2.6799	0.157	0.5268	-1.147	H1-L:0.26;H2-L1:0.26
	2	2.9225	424.24	0.008	1.259	0.2492	-1.738	H-L1:0.927	2.8611	1.278	0.2443	-1.673	H-L:0.839;H1-L2:0.022
	3	2.9869	415.09	0.002	0.215	0.3935	-1.281	H2-L:0.458;H1-L1:0.458	2.8611	1.279	0.2425	-1.733	H-L1:0.839;H2-L2:0.022
	4	3.0362	408.35	0	0.226	0.3968	-1.362	H1-L:0.439;H2-L1:0.434	2.8873	0.215	0.2899	-1.281	H2-L:0.319;H1-L1:0.319
	5	3.0397	407.89	0.107	0.303	0.3887	-2.107	H1-L1:0.371;H2-L:0.369	2.8873	0.215	0.2898	-1.282	H2-L1:0.319;H1-L:0.319
	6	3.0397	407.88	0.107	0.303	0.3887	-2.122	H2-L1:0.374;H1-L:0.366	2.9109	0.19	0.428	-1.452	H1-L1:0.337;H2-L:0.336

Table S4. TD-DFT calculated energy level diagram, the corresponding SOC constants, and the calculated facile RISC channels of S-CNDF-S-*t*Cz, S-CNDF-D-*t*Cz, and T-CNDF-T-*t*Cz

Compound	S_n/T_n	eV-S	eV-T	SOC-S_0	SOC-S_1	SOC-S_2	SOC-S_3	SOC-S_4	SOC-S_5	SOC-S_6	Facile RISC Channel (both $\Delta E_{ST} \leq 0.37$ eV, and SOC $\geq$0.3 cm^{-1})[1]
S-CNDF-S-*t*Cz	1	3.0781	2.9496	0.72008	0.2422	0.2432	0.64449	0.4458	0.40968	0.537	None
	2	3.4765	2.9835	0.49176	0.22432	0.3995	0.69328	0.34677	0.3036	0.2147	
	3	4.0306	2.9889	1.0075	0.16157	0.5601	0.72438	0.63591	0.47147	0.48147	
	4	4.1258	3.33	0.58254	0.06905	0.0237	0.12545	0.51805	0.25656	0.32644	
	5	4.3482	3.4032	0.62359	0.41191	0.1674	0.21193	0.254	0.29387	0.2493	
	6	4.4136	3.5145	0.76856	0.50391	0.0544	0.13885	0.53787	0.34857	0.09792	
S-CNDF-D-*t*Cz	1	3.1333	2.9862	1.02567	0.09269	0.5122	0.44634	0.27179	0.59261	0.7562	$T_5 \rightarrow S_1$, $T_1 \rightarrow S_2$, $T_2 \rightarrow S_2$, $T_3 \rightarrow S_2$, $T_4 \rightarrow S_2$
	2	3.2206	3.0016	0.7716	0.27687	0.3603	0.18203	0.40863	0.33766	0.5859	
	3	3.6256	3.0184	0.81935	0.12993	0.5223	0.30434	0.0882	0.33426	0.3225	
	4	3.6704	3.0415	0.58706	0.26977	0.49	0.34482	0.20583	0.11315	0.3888	
	5	3.8921	3.1037	0.64395	0.62332	0.0861	0.3797	0.32664	0.17866	0.2997	
	6	4.0054	3.3002	0.7567	0.10988	0.2685	0.04834	0.06665	0.45473	0.03292	
T-CNDF-T-*t*Cz	1	2.9225	2.6799	0.24356	0.2678	0.2678	0.53478	0.04649	0.39084	0.39111	$T_1 \rightarrow S_3$, $T_6 \rightarrow S_4$, $T_1 \rightarrow S_5$, $T_3 \rightarrow S_5$, $T_5 \rightarrow S_5$, $T_1 \rightarrow S_6$, $T_2 \rightarrow S_6$, $T_4 \rightarrow S_6$
	2	2.9225	2.8611	3.02453	0.0227	0.1014	0.06894	0.05556	0.22153	0.32603	
	3	2.9869	2.8611	3.02356	0.10167	0.0227	0.06877	0.05463	0.32646	0.22201	
	4	3.0362	2.8873	0.37562	0.2258	0.0825	0.06773	0.1356	0.04208	1.00738	
	5	3.0397	2.8873	0.38002	0.08224	0.2258	0.06773	0.13522	1.00747	0.04303	
	6	3.0397	2.9109	0.50649	0.05835	0.0584	0.0438	0.98335	0.03743	0.03757	

III. References

1. (a) Chen, R.; Tang, Y.; Wan, Y.; Chen, T.; Zheng, C.; Qi, Y.; Cheng, Y.; Huang, W., Promoting Singlet/triplet Exciton Transformation in Organic Optoelectronic Molecules: Role of Excited State Transition Configuration. *Scientific Reports* **2017,** *7* (1), 6225; (b) Tao, Y.; Chen, R.; Li, H.; Yuan, J.; Wan, Y.; Jiang, H.; Chen, C.; Si, Y.; Zheng, C.; Yang, B.; Xing, G.; Huang, W., Resonance-Activated Spin-Flipping for Efficient Organic Ultralong Room-Temperature Phosphorescence. *Advanced Materials* **2018,** *30* (44), 1803856.
